# Association of Perioperative Colchicine Use with Morbidity and Inflammation in Heart Valve Surgery

**DOI:** 10.3390/medicina62101917

**Published:** 2026-10-04

**Authors:** Mehmet Burak Gulcan, Mehmet Ali Kaygın, Aycan Mutlu Yaganoglu, Ziya Yıldız, Abdulselam Karaduman, Suleyman Aycan, Ozgur Dag

**Affiliations:** 1 Department of Cardiovascular Surgery, Ankara Yıldırım Beyazıt University Yenimahalle Training and Research Hospital, 06370 Ankara, Türkiye; 2 Department of Cardiovascular Surgery, Erzurum City Hospital, 25240 Erzurum, Türkiye; 3 Department of Zootechnics, Faculty of Agriculture, Ataturk University, 25240 Erzurum, Türkiye; 4 Department of Congenital Cardiovascular Surgery, Mehmet Akif Ersoy Chest and Cardiovascular Surgery Research and Training Hospital, 34303 Istanbul, Türkiye

**Keywords:** colchicine, cardiac surgery, valvular heart disease, cardiopulmonary bypass, inflammation, postoperative morbidity

## Abstract

*Background and Objectives*: Valvular heart disease (VHD) is a major cause of morbidity and mortality worldwide, and valve replacement surgery performed under cardiopulmonary bypass (CPB) with full sternotomy remains an important treatment modality. Colchicine has well-established anti-inflammatory effects in ischemic heart disease and pericardial disease, but its association with postoperative morbidity in patients undergoing valvular heart surgery has not been well studied. This study aimed to investigate the association of perioperative colchicine use with clinical morbidity and inflammatory parameters in patients undergoing valvular heart surgery with CPB. *Materials and Methods*: In this retrospective study, 82 patients who underwent elective on-pump valvular heart surgery through full sternotomy were divided into a control group (*n* = 42) who did not receive colchicine and a colchicine group (*n* = 40) who received oral colchicine (0.5 mg once daily) for three days before and ten days after surgery. Demographic, clinical, and laboratory data, including length of hospital and intensive care unit (ICU) stay, transfusion requirements, sternal wound complication, surgical re-exploration, and inflammatory parameters on postoperative day 5 (C-reactive protein [CRP], white blood cell count, albumin, platelet-to-lymphocyte ratio [PLR], and neutrophil-to-lymphocyte ratio [NLR]), were compared between the groups. *Results*: Length of ICU stay was significantly shorter in the colchicine group than in the control group (4.1 ± 1.6 vs. 5.3 ± 2.4 days; *p* = 0.009), and fresh frozen plasma transfusion requirement was significantly lower in the colchicine group (4.2 ± 2.1 vs. 6.9 ± 3.3 units; *p* < 0.001). Total hospital stay, erythrocyte transfusion requirement, and inflammatory parameters (CRP, white blood cell count, NLR, PLR, and albumin) did not differ significantly between the two groups (all *p* > 0.05). *Conclusions*: Perioperative colchicine was associated with a shorter ICU stay and a reduced requirement for fresh frozen plasma transfusion in patients undergoing valvular heart surgery with CPB, suggesting that colchicine may have a potential role in this surgical population. After correction for multiple comparisons, the difference in ICU length of stay was no longer statistically significant. Larger prospective studies are warranted to confirm these findings.

## 1. Introduction

In 2021, tens of millions of people worldwide were reported to have valvular heart disease (VHD) [1]. VHD has become an important public health problem, affecting morbidity, mortality, and quality of life, particularly in the elderly population [1]. Treatment options range from watchful waiting to open-heart surgery, including valve repair and replacement, depending on the decision of the heart team [2,3]. A substantial proportion of patients with VHD require surgical intervention [4]. Although the number of minimally invasive procedures is increasing, standard median sternotomy with cardiopulmonary bypass (CPB) still accounts for the largest proportion of VHD operations [5]. Accordingly, we conducted our study on patients who underwent valvular surgery under CPB with full sternotomy. We investigated clinical morbidity factors such as total length of hospital stay, length of intensive care unit stay, number of erythrocyte and fresh frozen plasma transfusions, wound infection, and surgical revision. In addition, inflammatory parameters on postoperative day 5 were assessed, including C-reactive protein (CRP), white blood cell count, albumin, and complete blood count (CBC)-based parameters such as the platelet-to-lymphocyte ratio (PLR) and neutrophil-to-lymphocyte ratio (NLR). The literature supports the use of CBC-derived inflammatory parameters in evaluating disease progression [6]. In this study, we compared patients who did and did not receive colchicine. Following the Low-Dose Colchicine (LoDoCo) [7], Colchicine Cardiovascular Outcomes Trial (COLCOT) [8], and LoDoCo2 [9] trials, colchicine has become a part of cardiovascular medicine, in addition to its conventional role in the treatment of pericarditis. However, the role of colchicine in VHD and valvular heart surgery is not as well established as in ischemic heart disease. On the other hand, CPB related inflammatory response remain its importance with contributions of cytokines, systemic inflammatory respond, immunoglobulin reaction [10,11]. Colchicine may attenuate CPB-related inflammation in patients undergoing valvular heart surgery. In this regard, we believe our findings may add to the limited literature on this topic. Our aim was to contribute new evidence in this area.

## 2. Materials and Methods

This retrospective clinical study was approved by the Scientific Research Ethics Committee of the University of Health Sciences, Erzurum City Hospital (decision no. 2025/05-133) and was conducted in accordance with the ethical principles outlined in the Declaration of Helsinki. The study included patients who underwent elective on-pump valvular heart surgery performed by the same surgical, anesthesia, perfusion, and intensive care unit (ICU) teams.

A total of 42 patients who underwent valvular heart surgery during the one-year period from October 2021 to October 2022 were consecutively selected to constitute the control group. These patients did not receive perioperative colchicine treatment. This group included all eligible patients treated during this period. Beginning in September 2023, based on contemporary guidelines for chronic and acute ischemic heart disease [12,13], colchicine was incorporated into the routine perioperative medical treatment of patients undergoing open-heart surgery in the Department of Cardiovascular Surgery at Erzurum City Hospital. Colchicine was administered orally at a dose of 0.5 mg once daily for three days before and ten days after surgery. A total of 40 patients who underwent valvular heart surgery during the one-year period from January 2024 to January 2025 were consecutively selected to constitute the colchicine group. This group included all eligible patients treated during this period.

Demographic, clinical, and laboratory data were retrospectively obtained from archived medical records and the hospital’s electronic information system. The collected variables included age, sex, total length of hospital stay, length of ICU stay, number of units of erythrocyte suspension and fresh frozen plasma transfused, surgical wound complication, and the need for surgical revision. Routinely measured inflammatory and laboratory parameters obtained on postoperative day 5 were also recorded, including C-reactive protein (CRP), white blood cell count, serum albumin level, platelet-to-lymphocyte ratio (PLR), and neutrophil-to-lymphocyte ratio (NLR).

Patients who underwent minimally invasive procedures, robotic valve surgery, or emergency surgery were excluded. Additional exclusion criteria included in-hospital mortality, renal failure, hepatic failure, the use of an intra-aortic balloon pump (IABP) or extracorporeal membrane oxygenation (ECMO), and the need for an additional surgical procedure because of cannulation-related complications. Patients requiring prolonged postoperative mechanical ventilation were also excluded. After the eligibility criteria were applied, all consecutive eligible patients were identified from the hospital’s archived medical records and electronic database.

All valvular heart surgery procedures were performed through a median sternotomy under general anesthesia by the same surgical and anesthesia teams. Cardiopulmonary bypass was established in all patients using standard aortic and bicaval cannulation. Myocardial protection was achieved using antegrade administration of Del Nido cardioplegia directly into the aortic root. All procedures were performed by the same perfusion team. Following surgery, all patients were transferred to the cardiovascular surgery ICU and were extubated on the day of surgery. Patients who could not be extubated on the day of surgery were excluded. In patients who developed colchicine-related adverse effects, such as diarrhea, nausea, or abdominal pain, colchicine was routinely discontinued, and these patients were also excluded. After mechanical valve replacement, lifelong anticoagulation was provided with an oral vitamin K antagonist (VKA), with a target INR of 2.5. After valve repair or bioprosthetic valve replacement, patients routinely received effective VKA therapy for the first 3 months, followed by lifelong acetylsalicylic acid (ASA). The term “sternal wound complication” encompasses a wide spectrum of wound findings, ranging from minor drainage related to fat necrosis to the need for surgical revision. All documented wound issues were included in this category based on the daily follow-up notes in the medical records. All patients were managed in the ICU according to a standardized institutional protocol with respect to transfusion thresholds, blood conservation strategies, and postoperative care.

In the colchicine group (*n* = 40), isolated aortic valve replacement (AVR) was the most common procedure, performed in 16 patients (40.0%). Mitral valve replacement combined with tricuspid valve repair (MVR + TR repair) was performed in 9 patients (22.5%), and isolated mitral valve replacement (MVR) in 4 patients (10.0%). Isolated mitral valve repair was performed in 3 patients (7.5%), and double valve replacement (AVR + MVR) in 2 patients (5.0%). The remaining 6 patients (15.0%) underwent more complex or combined procedures, each occurring once: MVR with tricuspid annuloplasty (De Vega) with or without ablation, a triple-valve procedure, combined AVR + MVR + tricuspid repair, combined mitral and tricuspid valve repair, and MVR with aortic valve repair.

In the control group (*n* = 42), isolated MVR and double valve replacement (AVR + MVR) were the most frequent procedures, each performed in 9 patients (21.4%). MVR combined with tricuspid repair was performed in 7 patients (16.7%), and isolated AVR in 7 patients (16.7%). A triple-valve procedure was performed in 3 patients (7.1%), while isolated aortic valve repair and isolated mitral valve repair were each performed in 2 patients (4.8%). The remaining 3 patients (7.1%) underwent combined mitral and tricuspid valve repair, and isolated tricuspid valve repair.

### Statistical Analysis

Statistical analyses were performed using IBM SPSS Statistics, version 26.0 (IBM Corp., Armonk, NY, USA). The normality of continuous variables was assessed within each group using the Shapiro–Wilk test. Because several variables deviated from a normal distribution, between-group comparisons were performed using both Welch’s *t*-test and the two-sided Mann–Whitney U test. To account for multiple comparisons, the Holm correction was applied to the ten between-group Welch comparisons. Continuous variables were expressed as mean ± standard deviation (SD). Comparisons of continuous variables between two independent groups were performed using the independent samples *t*-test. Levene’s test was used to assess the homogeneity of variances between groups; when this assumption was violated (Levene’s *p* < 0.05), the Welch-corrected *t*-test (equal variances not assumed) was applied instead of the standard Student’s *t*-test. Independent group comparisons were performed according to colchicine use (colchicine group vs. control group), sex, presence of a sternal wound complication (SWC), and requirement for surgical re-exploration. Associations among continuous clinical and laboratory parameters were evaluated using Pearson correlation analysis. A two-tailed *p*-value of less than 0.05 was considered statistically significant for all analyses.

## 3. Results

A total of 82 patients were included in the analysis. Of these, 42 patients (51.2%) did not receive colchicine (control group) and 40 patients (48.8%) received colchicine (colchicine group). The overall cohort comprised 44 men (53.7%) and 38 women (46.3%). A sternal wound complication developed in 24 patients (29.3%), while 58 patients (70.7%) had no evidence of SWC. Surgical re-exploration was required in 6 patients (7.3%), whereas 76 patients (92.7%) did not require re-exploration.

The primary treatment-group comparisons are shown in Table 1. FFP transfusion was lower in the colchicine group than in controls (4.2 ± 2.1 vs. 6.9 ± 3.3 units; Welch *p* < 0.001; Holm-adjusted *p* < 0.001). ICU stay was also shorter (4.1 ± 1.6 vs. 5.3 ± 2.4 days; Welch *p* = 0.009), but this comparison did not meet the Holm-adjusted threshold (*p* = 0.079). None of the other eight comparisons, including age, hospital stay, RBC transfusion, CRP, WBC, NLR, PLR, and albumin, was significant after Holm correction. The Mann–Whitney results for CRP (*p* = 0.014) and PLR (*p* = 0.001) differed from the corresponding Welch/Holm results; these rank-based sensitivity results do not change the primary interpretation and may reflect distributional differences.

Effect-size estimates provide context for the group differences (control minus colchicine). ICU stay differed by 1.2 days (95% CI: 0.3–2.1; Cohen’s d = 0.59, 95% CI: 0.14–1.03), although its Holm-adjusted *p*-value was 0.079. FFP use differed by 2.7 units (95% CI: 1.5–3.9; Cohen’s d = 0.97, 95% CI: 0.51–1.43; Holm-adjusted *p* < 0.001). The CRP mean difference was −22.0 mg/L (95% CI: −44.2 to 0.2; Cohen’s d = −0.44, 95% CI: −0.87 to 0.00; Holm-adjusted *p* = 0.414). The remaining group comparisons also did not meet the Holm-adjusted threshold. These unadjusted effect estimates do not account for differences in surgical procedure or other confounders.

When patients were stratified by sex (Table 2), no statistically significant differences were observed between men (*n* = 44) and women (*n* = 38) for age (*p* = 0.964), length of ICU stay (*p* = 0.778), total hospital stay (Welch-corrected, *p* = 0.737), RBC transfusion (Welch-corrected, *p* = 0.929), FFP transfusion (*p* = 0.485), CRP (*p* = 0.113), WBC count (*p* = 0.740), NLR (*p* = 0.555), or PLR (*p* = 0.893). Serum albumin values were numerically higher in women (32.7 ± 3.3 g/L) than in men (31.1 ± 3.9 g/L), although the difference was not statistically significant (t(80) = −1.924, *p* = 0.058). These comparisons should be regarded as exploratory.

Patients who developed a sternal wound complication (SWC; *n* = 24) were compared with those who did not (*n* = 58) (Table 3). Total hospital stay was significantly longer in patients with SWC (31.8 ± 8.8 days) than in those without (27.1 ± 8.6 days; t(80) = −2.226, *p* = 0.029). RBC transfusion requirement was significantly higher in the SWC group (6.5 ± 3.1 units) than in the non-SWC group (4.2 ± 1.9 units; Welch-corrected t(30.4) = −3.503, *p* = 0.001). FFP transfusion was also significantly greater in patients with SWC (6.6 ± 2.8 units) than in those without (5.1 ± 3.1 units; t(80) = −2.030, *p* = 0.044). CRP levels were significantly higher among patients with SWC (100.7 ± 66.4 mg/L) than among those without (63.5 ± 39.6 mg/L; t(80) = −3.141, *p* = 0.002). Age (Welch-corrected, *p* = 0.071), length of ICU stay (Welch-corrected, *p* = 0.052), WBC count (*p* = 0.195), NLR (*p* = 0.727), PLR (*p* = 0.829), and albumin (*p* = 0.066) did not differ significantly between groups. These comparisons should be regarded as exploratory.

Patients who required surgical re-exploration (*n* = 6) were compared with those who did not (*n* = 76) (Table 4). FFP transfusion was significantly higher in patients requiring re-exploration (8.0 ± 3.0 units) than in those who did not (5.4 ± 3.0 units; t(80) = −2.047, *p* = 0.044). RBC transfusion was numerically higher in the re-exploration group (8.7 ± 4.1 units) than in the non-re-exploration group (4.5 ± 2.1 units); however, because Levene’s test indicated markedly unequal variances (*p* = 0.003) and the re-exploration subgroup was small (*n* = 6), the Welch-corrected comparison did not reach statistical significance (t(5.2) = −2.451, *p* = 0.056). Age (*p* = 0.961), length of ICU stay (*p* = 0.194), total hospital stay (*p* = 0.527), CRP (*p* = 0.496), WBC count (*p* = 0.985), NLR (*p* = 0.727), PLR (*p* = 0.829), and albumin (*p* = 0.066) did not differ significantly between groups. Given the marked imbalance in group sizes, these comparisons should be regarded as exploratory.

Pearson correlation analysis among clinical and laboratory parameters for the entire cohort (*n* = 82) is summarized in Table 5. Length of ICU stay correlated positively with RBC transfusion (r = 0.420, *p* < 0.001), FFP transfusion (r = 0.340, *p* = 0.002), and total hospital stay (r = 0.298, *p* = 0.007), and correlated negatively with serum albumin (r = −0.253, *p* = 0.022). Total hospital stay was also negatively correlated with albumin (r = −0.280, *p* = 0.011). RBC transfusion correlated positively with FFP transfusion (r = 0.384, *p* < 0.001) and WBC count (r = 0.288, *p* = 0.009), and negatively with albumin (r = −0.263, *p* = 0.017). FFP transfusion correlated positively with WBC count (r = 0.252, *p* = 0.022). CRP correlated negatively with albumin (r = −0.251, *p* = 0.023). WBC count correlated positively with NLR (r = 0.295, *p* = 0.007), and NLR showed a strong positive correlation with PLR (r = 0.765, *p* < 0.001). No other correlations reached statistical significance.

Shapiro–Wilk tests were performed within each group in Table 6. The primary treatment-group analysis uses two-sided Welch tests and Holm correction for the ten comparisons in this table. Two-sided Mann–Whitney U *p*-values are sensitivity analyses for distributional differences and do not determine the primary conclusions. Exploratory age- and sex-adjusted OLS results for ICU stay and FFP units are presented in Table 7. Non-significant Shapiro–Wilk *p*-values do not establish normality.

Exploratory adjusted models (Table 7) included all 82 patients. Relative to controls, the colchicine group had an estimated ICU stay difference of −1.25 days (HC3 95% CI: −2.26 to −0.24; *p* = 0.016) and an FFP difference of −2.95 units (95% CI: −4.20 to −1.70; *p* < 0.001), after adjustment for age and sex. These exploratory *p*-values were not corrected for multiplicity and cannot account for differences in surgery type between study periods.

## 4. Limitations

Because of the retrospective design, some baseline surgical and clinical data were not available. In addition, prognostic risk scores such as EuroSCORE II were not available, which is another limitation of the study. Most importantly, the exclusion of patients who died in hospital, required prolonged mechanical ventilation, or needed IABP/ECMO support may have introduced post-treatment selection bias, which is acknowledged as a major limitation of the present study. Furthermore, the retrospective design, the small sample size, the use of non-contemporary historical controls, potential selection bias introduced by postoperative exclusion criteria, the absence of multivariable adjustment, unmeasured differences in surgical complexity, the lack of baseline inflammatory marker values, the risk of multiple testing, and the inability to establish causality are additional limitations of this study. In addition, the exclusion of patients who developed colchicine-related adverse effects and the considerable heterogeneity in the types of surgical procedures performed during the two study periods, absence of multivariable adjustment constitute further limitations of this study.

## 5. Discussion

Colchicine is an ancient drug derived from the plant Colchicum autumnale, a type of flowering plant [14]. Colchicine has been used to treat swelling, pain, and gout since antiquity [14]. Currently, colchicine is used to treat a wide range of inflammatory conditions, including gout, Familial Mediterranean Fever (FMF), and Behçet’s disease, as well as cardiovascular diseases such as pericarditis and coronary artery disease [15].

Colchicine exerts its anti-inflammatory effects through inhibition of tubulin polymerization [16]. Microtubule polymerization controls many intracellular pathways, including organelle and vesicle transport, cell migration, and cytokine and chemokine secretion [15,16]. Colchicine also inhibits neutrophil chemotaxis, adhesion, mobilization, and recruitment [15]. Furthermore, colchicine inhibits interleukin-1β (IL-1β) release and exerts antifibrotic effects [15].

The first use of colchicine in cardiovascular medicine was in pericardial disease. In the 1990s, the benefits of colchicine in recurrent pericarditis were clearly demonstrated [17,18]. Colchicine was subsequently found to be beneficial in post-pericardiotomy syndrome [19]. This was important because postoperative pericardial effusion may occur in patients who undergo open-heart surgery. The Colchicine for the Prevention of the Post-Pericardiotomy Syndrome (COPPS) trial demonstrated that colchicine was effective in preventing post-pericardiotomy syndrome (PPS) [20]. Subsequently, the COPPS-2 trial was conducted [21]. In addition to post-pericardiotomy syndrome, the COPPS-2 trial evaluated postoperative atrial fibrillation and postoperative pericardial or pleural effusion [21]. Its results were similar to those of COPPS with respect to PPS reduction, although colchicine did not prevent postoperative atrial fibrillation or pericardial/pleural effusion [21]. Currently, both European and American guidelines recommend colchicine as an important treatment modality for pericardial diseases [22,23]. In addition, colchicine plays a crucial role in the management of PPS according to the ESC guidelines [22]. This is another reason for our use of colchicine in the perioperative period of open-heart surgery.

In the COP-AF study, colchicine did not significantly reduce atrial fibrillation (AF) or myocardial injury compared with placebo after major non-cardiac thoracic surgery [24]. However, colchicine was associated with increased rates of diarrhea in this study [24]. In contrast, Shvartz et al. [25] reported contrasting results regarding AF prevention after cardiac surgery in the early postoperative period. Their study included both coronary and valvular surgery patients [25]. Tabbalat et al. [26] had previously investigated the same topic, and their results showed that colchicine had no benefit in postoperative AF prevention, similar to the COP-AF study [24].

The Colchicine and Inflammation in Aortic Stenosis (CHIANTI) trial is investigating whether colchicine may have a role in the treatment of valvular disease [27]. The trial is based on the hypothesis that low-dose colchicine may limit disease progression, particularly in moderate aortic stenosis [27]. Rifaie et al. [28] investigated the effects of low-dose colchicine in chronic rheumatic valvular disease. They found that low-dose colchicine was associated with lower levels of inflammatory markers such as IL-6 and CRP [28]. We also investigated CRP levels; however, our study was conducted only in valvular surgery patients, and we did not find any statistically significant difference between the groups. Ryffel et al. [29] investigated the effects of colchicine in TAVR patients. Although their results suggested that periprocedural colchicine use had benefits with respect to arrhythmia development and leaflet thrombosis, the study was terminated early because of an unexpectedly high stroke rate [29]. Their study might have contributed more if it had not been terminated prematurely. In addition, our study differs from theirs in terms of surgical approach. Pan et al. [30] reported that colchicine was associated with improved myocardial protection in non-CABG open-heart surgery. They evaluated inflammatory and myocardial injury parameters such as troponin, CK-MB, IL-6, and procalcitonin [30]. In contrast, we limited our study to valvular heart surgery. We also focused on morbidity factors related to open-heart surgery, such as total length of hospital stay, length of intensive care unit stay, number of erythrocyte and fresh frozen plasma transfusions, age, sex, wound infection, and surgical revision.

CPB-related inflammation is triggered by multiple underlying mechanisms [10,11]. In particular, cytokines such as IL-6, IL-8, and IL-10 have been associated with CPB-related inflammation [10]. Adhesion molecules, such as selectins, integrins, and members of the immunoglobulin superfamily, also contribute to this inflammatory response [11]. In addition, transfusion is associated with postoperative inflammation in cardiac surgery [31]. Colchicine may have a role to attenuate inflammation. Regarding the impact of anti-inflammatory drugs on patient outcomes, steroid use has been associated with shorter ICU stays and fewer respiratory and infectious complications [32]. We hypothesize that colchicine might attenuate CPB-induced inflammation, which could, in turn, contribute to reduced blood product requirements and a more favorable ICU course. However, this hypothesis requires confirmation in future prospective studies.

## 6. Conclusions

Perioperative colchicine use was associated with a shorter length of intensive care unit stay and a lower requirement for fresh frozen plasma transfusion in patients undergoing valvular heart surgery with cardiopulmonary bypass in our retrospective study. In addition, after correction for multiple comparisons, the difference in ICU length of stay was no longer statistically significant. These findings may contribute to the literature on the valvular surgical population, which has received far less attention than the established indications of colchicine in ischemic heart disease and pericardial disease. To our knowledge, this is among the first studies to report such an association specifically in valvular heart surgery, and it may support a potential role for colchicine in reducing perioperative morbidity in this setting. Larger prospective, randomized studies are needed to confirm these findings and to clarify the optimal timing and dosing of colchicine in valvular surgery.

## Figures and Tables

**Table 1:** Colchicine vs. Control Group.

Parameter	Control Group (*n* = 42) Mean ± SD	Colchicine Group (*n* = 40) Mean ± SD	Welch *p*-Value (Unadjusted)	Holm-Adjusted *p*-Value
Age (years)	56.6 ± 13.2	57.3 ± 13.9	0.821	1.000
Length of ICU stay (days)	5.3 ± 2.4	4.1 ± 1.6	0.009	0.079
Total hospital stay (days)	28.8 ± 8.7	28.1 ± 9.1	0.728	1.000
RBC transfusion (units)	5.0 ± 2.3	4.7 ± 2.8	0.685	1.000
FFP transfusion (units)	6.9 ± 3.3	4.2 ± 2.1	<0.001	<0.001
CRP (mg/L)	63.6 ± 52.4	85.6 ± 48.6	0.052	0.414
WBC (×10^9^/L)	10.8 ± 4.5	9.9 ± 4.3	0.366	1.000
NLR	6.93 ± 8.43	6.72 ± 4.23	0.885	1.000
PLR	122.0 ± 170.6	172.3 ± 101.6	0.108	0.755
Albumin (g/L)	32.3 ± 3.7	31.4 ± 3.6	0.278	1.000

Table 1 compares treatment groups. Data are mean ± SD. Unadjusted *p*-values are from Welch’s two-sided *t*-test; Holm-adjusted *p*-values across all ten comparisons define the primary interpretation. ICU, intensive care unit; RBC, red blood cell; FFP, fresh frozen plasma; CRP, C-reactive protein; WBC, white blood cell count; NLR, neutrophil-to-lymphocyte ratio; PLR, platelet-to-lymphocyte ratio.

**Table 2:** Comparison by Sex.

Parameter	Men (*n* = 44) Mean ± SD	Women (*n* = 38) Mean ± SD	*p*-Value
Age (years)	56.9 ± 12.3	57.0 ± 14.8	0.964
Length of ICU stay (days)	4.7 ± 2.3	4.8 ± 2.0	0.778
Total hospital stay (days)	28.1 ± 7.4	28.8 ± 10.4	0.737 ^a^
RBC transfusion (units)	4.9 ± 3.1	4.8 ± 1.6	0.929 ^a^
FFP transfusion (units)	5.8 ± 3.3	5.3 ± 2.8	0.485
CRP (mg/L)	82.7 ± 60.0	64.7 ± 37.9	0.113
WBC (×10^9^/L)	10.2 ± 4.8	10.6 ± 3.9	0.740
NLR	6.42 ± 4.21	7.30 ± 8.75	0.555
PLR	144.5 ± 116.0	148.8 ± 170.0	0.893
Albumin (g/L)	31.1 ± 3.9	32.7 ± 3.3	0.058

Table 2 compares the same clinical and laboratory parameters according to patient sex, irrespective of colchicine use. Data are presented as mean ± SD and were compared using the independent samples *t*-test. ICU, intensive care unit; RBC, red blood cell; FFP, fresh frozen plasma; CRP, C-reactive protein; WBC, white blood cell count; NLR, neutrophil-to-lymphocyte ratio; PLR, platelet-to-lymphocyte ratio. No comparisons reached statistical significance (all *p* > 0.05). ᵃ Welch-corrected *t*-test applied because Levene’s test indicated unequal variances (*p* < 0.05).

**Table 3:** Comparison by Sternal Wound Complication (SWC) Status.

Parameter	No SWC (*n* = 58) Mean ± SD	SWC Present (*n* = 24) Mean ± SD	*p*-Value
Age (years)	55.6 ± 15.0	60.3 ± 8.1	0.071 ^a^
Length of ICU stay (days)	4.4 ± 1.5	5.7 ± 3.0	0.052 ^a^
Total hospital stay (days)	27.1 ± 8.6	31.8 ± 8.8	**0.029**
RBC transfusion (units)	4.2 ± 1.9	6.5 ± 3.1	**0.001 ^a^**
FFP transfusion (units)	5.1 ± 3.1	6.6 ± 2.8	**0.044**
CRP (mg/L)	63.5 ± 39.6	100.7 ± 66.4	**0.002**
WBC (×10^9^/L)	10.0 ± 4.7	11.4 ± 3.4	0.195
NLR	6.39 ± 7.43	7.88 ± 4.28	0.727
PLR	143.2 ± 153.0	154.5 ± 116.3	0.829
Albumin (g/L)	32.6 ± 3.7	30.1 ± 2.9	0.066

Table 3 compares clinical and laboratory parameters between patients with and without a sternal wound complication (SWC). Data are presented as mean ± SD and were compared using the independent samples *t*-test. Statistically significant values (*p* < 0.05) are shown in bold. SWC, sternal wound complication; ICU, intensive care unit; RBC, red blood cell; FFP, fresh frozen plasma; CRP, C-reactive protein; WBC, white blood cell count; NLR, neutrophil-to-lymphocyte ratio; PLR, platelet-to-lymphocyte ratio. ᵃ Welch-corrected *t*-test applied because Levene’s test indicated unequal variances (*p* < 0.05).

**Table 4:** Comparison by Surgical Re-exploration Status.

Parameter	No Re-Exploration (*n* = 76) Mean ± SD	Re-Exploration (*n* = 6) Mean ± SD	*p*-Value
Age (years)	56.9 ± 13.7	56.7 ± 9.5	0.961
Length of ICU stay (days)	4.7 ± 2.1	5.8 ± 2.1	0.194
Total hospital stay (days)	28.3 ± 8.9	30.7 ± 8.3	0.527
RBC transfusion (units)	4.5 ± 2.1	8.7 ± 4.1	0.056 ^a^
FFP transfusion (units)	5.4 ± 3.0	8.0 ± 3.0	**0.044**
CRP (mg/L)	73.3 ± 51.7	88.3 ± 50.8	0.496
WBC (×10^9^/L)	10.4 ± 4.5	10.4 ± 2.3	0.985
NLR	6.75 ± 6.83	7.75 ± 4.49	0.727
PLR	145.5 ± 146.1	158.8 ± 95.1	0.829
Albumin (g/L)	32.0 ± 3.7	29.2 ± 3.1	0.066

Table 4 compares clinical and laboratory parameters between patients who required surgical re-exploration and those who did not. Data are presented as mean ± SD and were compared using the independent samples *t*-test. Statistically significant values (*p* < 0.05) are shown in bold. The re-exploration subgroup was small (*n* = 6); results involving this subgroup should be interpreted with caution. ICU, intensive care unit; RBC, red blood cell; FFP, fresh frozen plasma; CRP, C-reactive protein; WBC, white blood cell count; NLR, neutrophil-to-lymphocyte ratio; PLR, platelet-to-lymphocyte ratio. ᵃ Welch-corrected *t*-test applied because Levene’s test indicated unequal variances (*p* < 0.05).

**Table 5:** Correlation Matrix (Pearson r).

Parameter	1	2	3	4	5	6	7	8	9
1. Length of ICU stay	—	0.298 **	0.420 **	0.340 **	−0.053	0.162	0.103	0.040	−0.253 *
2. Total hospital stay	0.298 **	—	0.062	0.002	0.168	0.152	0.090	0.096	−0.280 *
3. RBC transfusion	0.420 **	0.062	—	0.384 **	0.204	0.288 **	0.094	−0.012	−0.263 *
4. FFP transfusion	0.340 **	0.002	0.384 **	—	−0.119	0.252 *	0.056	−0.192	−0.102
5. CRP	−0.053	0.168	0.204	−0.119	—	0.018	0.074	0.150	−0.251 *
6. WBC	0.162	0.152	0.288 **	0.252 *	0.018	—	0.295 **	0.057	−0.056
7. NLR	0.103	0.090	0.094	0.056	0.074	0.295 **	—	0.765 **	−0.109
8. PLR	0.040	0.096	−0.012	−0.192	0.150	0.057	0.765 **	—	−0.032
9. Albumin	−0.253 *	−0.280 *	−0.263 *	−0.102	−0.251 *	−0.056	−0.109	−0.032	—

Table 5 shows Pearson correlation coefficients (r) among clinical and laboratory parameters for the entire cohort (*n* = 82); statistically significant coefficients (*p* < 0.05) are shown in bold. ICU, intensive care unit; RBC, red blood cell; FFP, fresh frozen plasma; CRP, C-reactive protein; WBC, white blood cell count; NLR, neutrophil-to-lymphocyte ratio; PLR, platelet-to-lymphocyte ratio. * *p* < 0.05; ** *p* < 0.01 (two-tailed).

**Table 6:** Group comparisons and distribution checks: colchicine vs. control group.

Parameter	Control Group (*n* = 42) Mean ± SD	Colchicine Group (*n* = 40) Mean ± SD	Shapiro–Wilk *p* (Control/Colchicine)	Welch *p*-Value	Holm-Adjusted *p*-Value	Mann–Whitney *p*-Value
Age (years)	56.6 ± 13.2	57.3 ± 13.9	0.010/0.840	0.821	1.000	1.000
Length of ICU stay (days)	5.3 ± 2.4	4.1 ± 1.6	<0.001/<0.001	0.009	0.079	0.006
Total hospital stay (days)	28.8 ± 8.7	28.1 ± 9.1	0.096/0.189	0.728	1.000	0.666
RBC transfusion (units)	5.0 ± 2.3	4.7 ± 2.8	0.003/<0.001	0.685	1.000	0.348
FFP transfusion (units)	6.9 ± 3.3	4.2 ± 2.1	<0.001/<0.001	<0.001	<0.001	<0.001
CRP (mg/L)	63.6 ± 52.4	85.6 ± 48.6	<0.001/0.180	0.052	0.414	0.014
WBC (×10^9^/L)	10.8 ± 4.5	9.9 ± 4.3	0.001/<0.001	0.366	1.000	0.260
NLR	6.93 ± 8.43	6.72 ± 4.23	<0.001/0.006	0.885	1.000	0.301
PLR	122.0 ± 170.6	172.3 ± 101.6	<0.00/0.023	0.108	0.755	0.001
Albumin (g/L)	32.3 ± 3.7	31.4 ± 3.6	0.588/0.454	0.278	1.000	0.235

Table 6 gives the complete distribution checks and sensitivity tests. Data are mean ± SD. Shapiro–Wilk *p*-values refer to control and colchicine groups, respectively. Welch *p*-values are unadjusted; the Holm column provides the primary *p*-values across ten comparisons, including age. Mann–Whitney *p*-values are sensitivity analyses and should not automatically be interpreted as tests of medians. ICU, intensive care unit; RBC, red blood cell; FFP, fresh frozen plasma; CRP, C-reactive protein; WBC, white blood cell count; NLR, neutrophil-to-lymphocyte ratio; PLR, platelet-to-lymphocyte ratio.

**Table 7:** Exploratory age- and sex-adjusted models for ICU stay and FFP transfusion.

Outcome	Predictor	Adjusted Coefficient	HC3 95% CI	*p*-Value
ICU stay (days)	Colchicine vs. control	−1.254	−2.264 to −0.245	0.016
ICU stay (days)	Age, per year	0.019	−0.004 to 0.043	0.107
ICU stay (days)	Female vs. male	−0.148	−1.157 to 0.862	0.772
FFP transfusion (units)	Colchicine vs. control	−2.949	−4.197 to −1.701	<0.001
FFP transfusion (units)	Age, per year	0.023	−0.010 to 0.057	0.172
FFP transfusion (units)	Female vs. male	−1.139	−2.387 to 0.109	0.073

Table 7 Ordinary least-squares regression, *n* = 82 per model. Coefficients are adjusted mean differences for the stated predictor, conditional on the other two covariates. HC3 robust standard errors and t-based 95% confidence intervals used 78 residual degrees of freedom. Reference groups: control and male. These models are exploratory and do not control for procedure type, operative complexity, or other perioperative confounders.

## Data Availability

The data supporting the findings of this study are available from the corresponding author upon reasonable request.
